# Supplementary figures and images for: GenoVi, an open-source automated circular genome visualizer for bacteria and archaea
Source: PLoS Comput Biol. 2023 Apr 4;19(4):e1010998. doi: 10.1371/journal.pcbi.1010998 (PMC10104344; doi:10.1371/journal.pcbi.1010998)

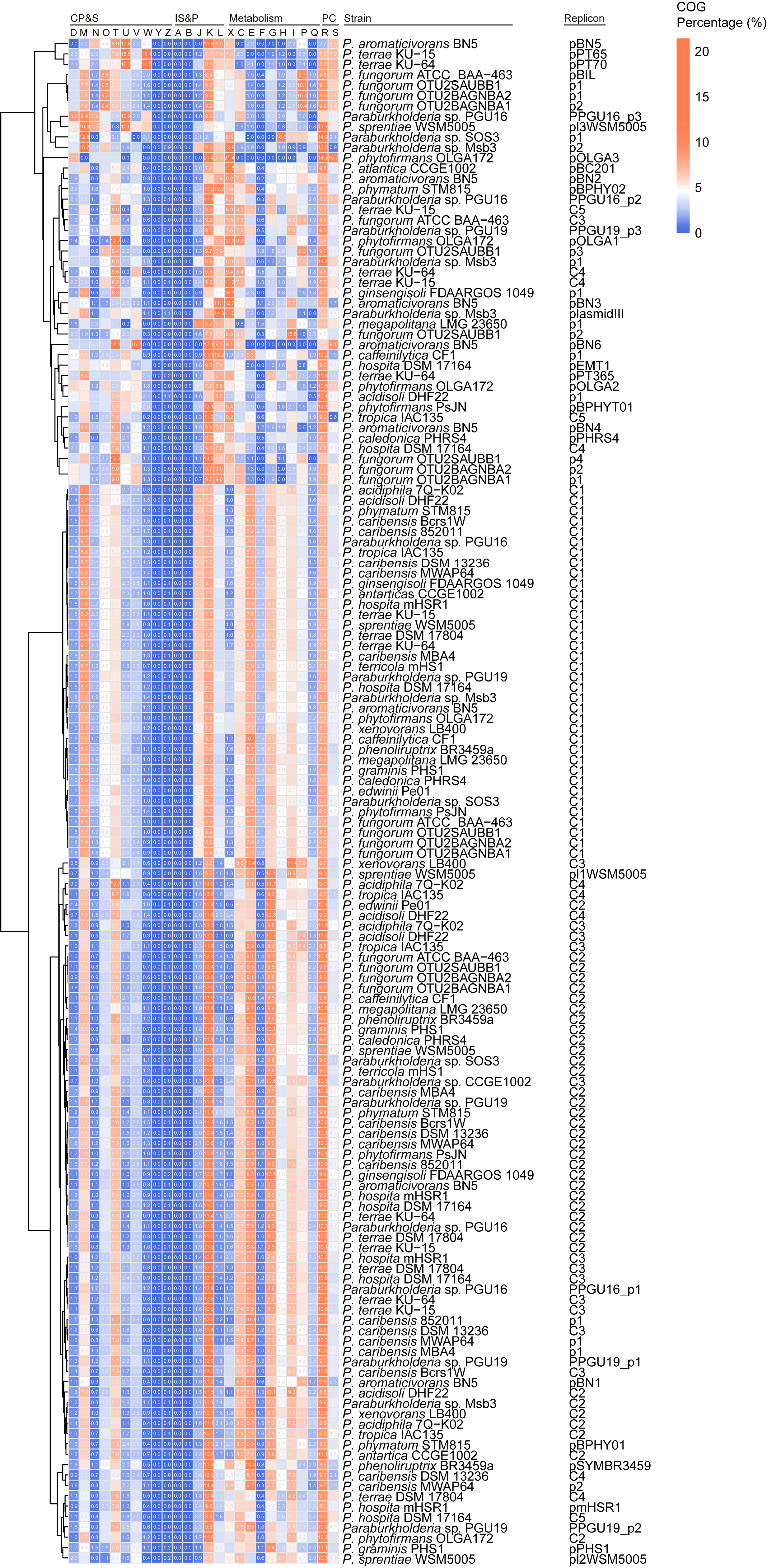

Supplement: S1 Fig — Hierarchical clustering of COG percentage shows three major groups in Paraburkholderia replicons. (TIF) [file pcbi.1010998.s001.tif]

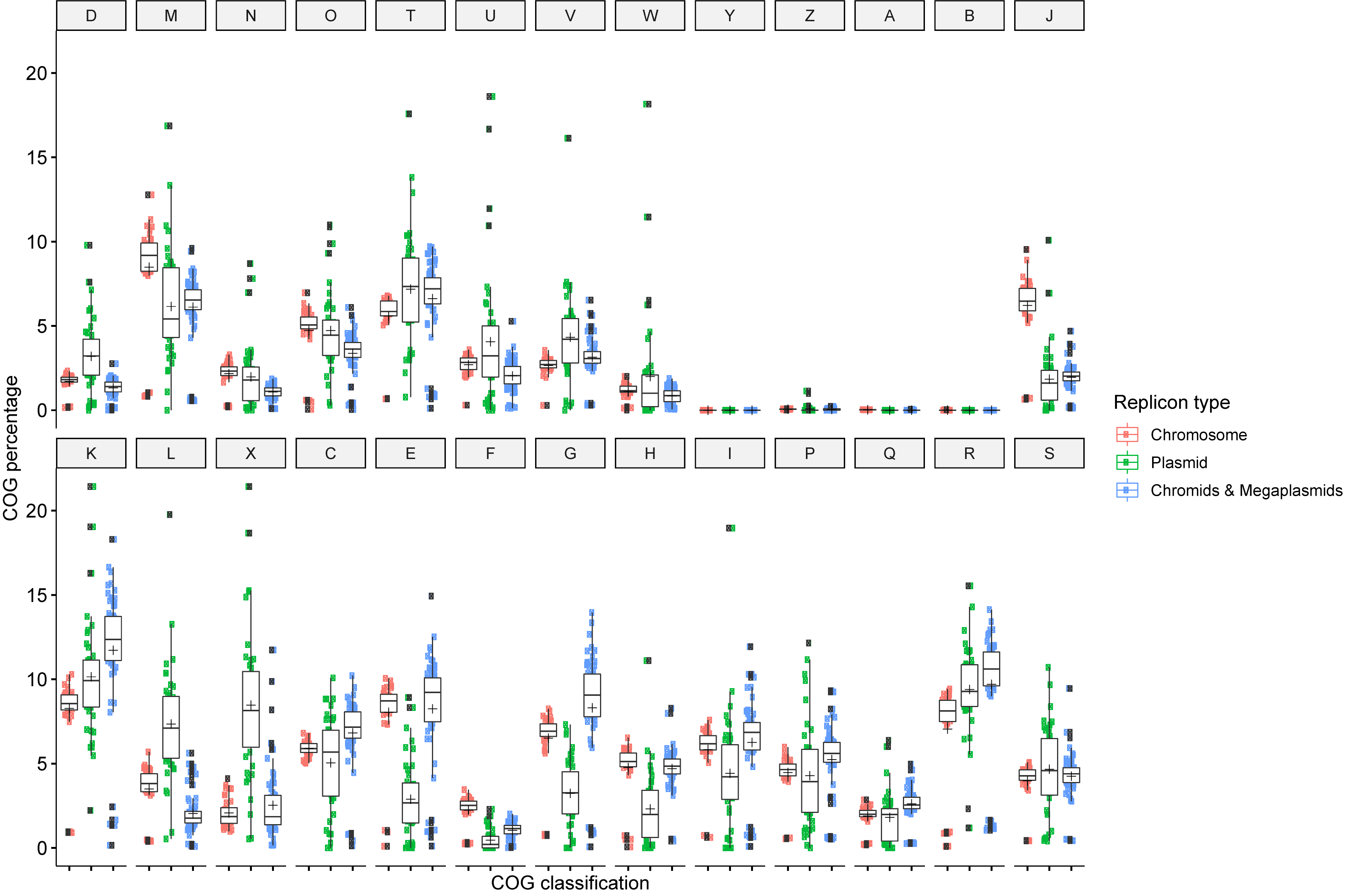

Supplement: S2 Fig — (TIF) [file pcbi.1010998.s002.tif]
