# Supplementary material for: GenoVi, an open-source automated circular genome visualizer for bacteria and archaea
Source: PLoS Comput Biol. 2023 Apr 4;19(4):e1010998. doi: 10.1371/journal.pcbi.1010998 (PMC10104344; doi:10.1371/journal.pcbi.1010998)
Supplement: S1 Table — (PDF) [file pcbi.1010998.s003.pdf]

S1 Table. General features of *Paraburkholderia* genomes used in this study

Organism Name	Accession N°	Size(Mb)	GC%	Replicons	CDS	Reference
P. edwinii Pe01	GCA_019428685.1	8.30	62.48	2	7073	[40]
Paraburkholderia sp. SOS3	GCA_001922345.1	7.50	63.30	3	6530	-
P. terricola mHS1	GCA_003330825.1	7.12	63.67	2	6225	[41]
P. caffeinilytica CF1	GCA_003368325.1	8.32	62.24	3	7174	[42]
P. megapolitana LMG 23650	GCA_007556815.1	7.63	62.04	3	6529	-
P. phytofirmans PsJN	GCA_000020125.1	8.21	62.32	3	7210	[32]
P. graminis PHS1	GCA_003330785.1	7.51	62.84	3	6548	[41]
P. caribensis MBA4	GCA_000522545.2	9.48	62.49	3	8195	[43]
P. ginsengisoli FDAARGOS_1049	GCA_016128195.1	6.60	63.54	3	5703	-
P. phenoliruptrix BR3459a	GCA_000300095.1	7.65	63.12	3	6559	[44]
P. acidiphila 7Q-K02	GCA_009789655.1	7.80	64.34	4	6869	[45]
Paraburkholderia sp. PGU19	GCA_013426915.1	11.25	61.71	5	9642	[46]
P. fungorum OTU2BAGNBA2	GCA_024072715.1	9.12	61.85	4	7944	-
P. terrae DSM 17804	GCA_002902925.1	10.06	61.94	4	8779	-
P. fungorum OTU2BAGNBA1	GCA_024072595.1	9.12	61.85	4	7948	-
Paraburkholderia sp. PGU16	GCA_013426895.1	9.47	62.38	5	8307	[46]
P. caribensis Bcrs1W	GCA_001611015.1	9.32	62.31	3	8071	[47]
P. caribensis 852011	GCA_013378095.1	8.47	62.80	3	7330	-
P. phymatum STM815	GCA_000020045.1	8.68	62.28	4	7486	[48]
P. acidisoli DHF22	GCA_009789675.1	7.95	65.41	5	6905	[45]
P. caledonica PHRS4	GCA_003330745.1	7.21	61.91	3	6131	[41]
P. caribensis DSM 13236	GCA_002902945.1	9.03	62.55	4	7821	-
P. caribensis MWAP64	GCA_001449005.1	9.03	62.55	4	7787	[49]
P. xenovorans LB400	GCA_000013645.1	9.73	62.63	3	8447	[30]
P. terrae KU-64	GCA_020885575.1	10.39	61.95	6	9123	[50]
P. fungorum ATCC BAA-463	GCA_000961515.1	9.06	61.77	4	7934	-
P. tropica IAC135/BECa 135	GCA_014171495.1	8.82	64.57	5	7601	[51]
P. hospita mHSR1	GCA_003330805.1	10.82	61.98	4	9442	[41]
P. phytofirmans OLGA172	GCA_001634365.1	8.57	60.85	5	7259	-
P. terrae KU-15	GCA_023169865.1	10.42	61.92	6	9123	[39]
P. atlantica CCGE1002	GCA_000092885.1	7.88	63.26	4	6869	[52]
P. sprentiae WSM5005	GCA_001865575.2	7.84	63.22	5	6812	[53]
P. hospita DSM 17164	GCA_002902965.1	11.53	61.81	6	10052	-
P. aromaticivorans BN5	GCA_002278075.1	8.91	62.93	8	7832	[31]
P. fungorum OTU2SAUBB1	GCA_024123895.1	9.36	61.73	6	8162	-
Paraburkholderia sp. Msb3	GCA_902459535.1	8.36	62.52	5	7441	-
