# Supplementary material for: GenoVi, an open-source automated circular genome visualizer for bacteria and archaea
Source: PLoS Comput Biol. 2023 Apr 4;19(4):e1010998. doi: 10.1371/journal.pcbi.1010998 (PMC10104344; doi:10.1371/journal.pcbi.1010998)
Supplement: S1 Text — (DOCX) [file pcbi.1010998.s004.docx]

**Supplementary methods**

***Paraburkholderia* comparative genomics analysis*:***

All the complete genomes of *Paraburkholderia* were retrieved using the Eutils software (db=nucleotide, rettype=gb,retmode=text; **S1 Table**). Complete genomes included a total of 147 replicons ranging from 22 kb to 4.94 Mb. To visualize and scale up all the replicons for a quick visual comparison, a directory including the 36 gbff files were given as an input for GenoVi using the complete status (-s complete). Therefore, each replicon was illustrated as a circular representation, and scaled up in relation to all the 147 replicons. Output tables for general features statistics and COG classifications per replicon were created automatically by GenoVi and used for further analyses.

The COG percentage distribution matrix was visualized and clustered to elucidate functional proportion of CDS per replicon using hclust (hclust, method= ward.D2, distance_method= euclidean) function of the pheatmaps package in R (Kolde, 2019) [1]. Size, GC content, tRNA and rRNA statistics from each class (Chromosomes; Chromids-Megaplasmids; Plasmids) were plotted as box-plots to show the data distribution among replicons using the ggplot2 package in R (Wickham, 2011) [2].

**References:**

1. Kolde, R. (2019). pheatmap: Pretty Heatmaps. R package.
2. Wickham, H. (2011). ggplot2. *Wiley interdisciplinary reviews: computational statistics*, *3*(2), 180-185.
