# Supplementary material for: GenoVi, an open-source automated circular genome visualizer for bacteria and archaea
Source: PLoS Comput Biol. 2023 Apr 4;19(4):e1010998. doi: 10.1371/journal.pcbi.1010998 (PMC10104344; doi:10.1371/journal.pcbi.1010998)
Supplement: Attachment — Submitted filename: Response to Reviewers.pdf [file pcbi.1010998.s006.pdf]

Response to Reviewers: PCOMPBIOL-D-22-01291

GenoVi, an open-source automated circular genome visualizer for bacteria and archaea

Andrés Cumsille, Roberto E. Durán, Andrea Rodríguez-Delherbe, Vicente Saona-Urmeneta, Beatriz Cámara, Michael Seeger, Mauricio Araya, Nicolás Jara, Carlos Buil-Aranda

I. Reviewer 1 (Dr. Michael J. Roach):

General Comments:

The manuscript describes a command line bioinformatics tool that is designed to take a genbank file and generate a Circos plot to display the GC content and skew, as well as ortholog positions using the COGs database. Creating Circos plots with the Circos program is a non-trivial task with a steep learning curve. The advantage of this tool is in the automated processing and rendering of Circos plots to display information that the authors believe would be most relevant to users when visualising bacterial genomes. There are other platforms and pipelines designed to help users create Circos plots, but each have their own strengths and weaknesses and having more options available to end users is always a good thing. The manuscript is well written, and the tool performs well.

Main concerns:

GenoVi is sold as a visualisation tool, and an example is given for how GenoVi can help distinguish replicons from plasmids in a bacterial genus. The example uses the COG annotations generated by GenoVi as input for a gene composition-based analysis, but it does not indicate whether or not the GenoVi visualisations themselves were at all helpful in this endeavour. My impression is that the findings of the example could be achieved by simply annotating the genome with DeepNOG and then preforming the subsequent analyses presented in the results. It would be beneficial to better highlight the usefulness of the visualisations that GenoVi creates.

Dear Dr. Michael J. Roach,

Thank you for the thorough and dedicated revision of our manuscript and software. We are kindly grateful for all your comments and raised concerns about GenoVi.
As stated by your revision, the previous version of the manuscript and software did not properly highlight the usefulness of GenoVi. The main example focused on *Paraburkholderia* mostly used output tables for its analysis, which could be obtained by other software without the main participation of the visualization.

To address the raised concerns by all the reviewers and editors, we added new features to GenoVi that modified the accompanying manuscript.

To improve the scope of GenoVi, additional features were incorporated into the new version. This included: enabling directories as an input (analyzed either as *-s draft* or *-s complete*), processing all the files independently and creating compilation tables that summarize the genomic features identified from each genome (general stats and COG categories); automatic generation of COG categories frequency per replicon or contig, and

heatmaps that illustrates the abundance of COG categories identified. Additionally, the user can specify which COG categories should be displayed in the ideogram, rather by directly specifying the categories (individually or as a functional group), or by specifying the N most abundant categories. With these new features, GenoVi demonstrates to be useful in various settings, which modified the examples in the manuscript. Currently, the analysis of *Paraburkholderia* multipartite genomes was reduced in extension, and other examples were incorporated to better illustrate the versatility of GenoVi.

Q1: Figure 2: It's impossible to tell what each of the tracks are. There are too many colours to simply rely on the colours alone to know what is what. I suggest using ideogram spacing and adding in labels for each track. http://www.circos.ca/documentation/tutorials/ideograms/spacing_breaks/lesson

A: Thank you for the suggestion. As recommended by the reviewer, we incorporated an optional flag that space the ideogram and include a label to each track (*-te; -- track explanation*). Additionally, the captions were enlarged, and we included some options for COGs categories display (using the *--cogs* argument). In this new version, the user can select which COG categories are displayed, facilitating its visualization. The example of *Rhodococcus* sp. H-CA8f illustrates both new features (**Fig 4**).

Minor comments and suggestions:

Q2: For draft genomes, how does GenoVi lay out the contigs (order and orientation) and can this interfere with GC skew calculations at the boundaries? For draft genomes, my understanding is that plasmid contigs and genome contigs are combined in the same circos plot. Is this correct? While this might not be ideal, there probably isn't a neat or easy solution for this. Maybe it would be best to just make sure this is clear in the documentation.

A: For draft genomes, GenoVi adds the order and orientation of contigs delivered by the initial genbank file. As stated by the reviewer this is not ideal, but there is no easy solution without comparison with a complete genome of a strain of the same species. By default parameters, GC skew calculation is performed using a 1000 bp window and using each contig independently. We have incorporated this information to the wiki and documentation of GenoVi.

Q3: """Interactive web platform""" – this would be a great future addition.

A: Thank you for the feedback. We will work on a web platform in the near future that will include additional features for interactive visualization, interaction with annotation files from other software, and customizable options.

Q4: """GenoVi is the first open-source tool that uses a unique input file …"""
Genbank files are not unique, which is good because you don't want unique (non-standard) file formats for your inputs.

A: Thank you for mentioning this. By saying "*unique*" we meant that it only requires 1 file as input to create a full representation, including a COG categories analysis, instead of requiring extra files (like. gff or other annotation files) to add that information. We rephrase this sentence for better understanding.

Q5: Running the Program:
Do you need bioconda and conda-forge channels when installing circos?

A: Circos is a base module included in a bioconda package, but it can also be installed separately by following the instructions on Circos website.

Q6: Are there plans to add this to bioconda? This would make the installation of GenoVI and Circos a one-step process.

A: Thank you for this recommendation. We are currently working to add GenoVi to bioconda for a clean installation. We hope for this installation method to be available during the next couple of months.

Q7: As the input file is always required, it could just be a bareword argument, e.g. genovi input.gb …

A: Thank you for the input. We considered including this suggestion, however, we decided to keep it for the sake of consistency, since the output file is optional.

Q8: Instead of specifying -status every time, consider having a -draft flag (and default to complete).
e.g. for complete genome:
genovi input.gb
e.g. for draft genome:
genovi input.gb -draft

A: Thanks for the suggestion. We considered that the user should be compelled to specify this flag, otherwise could lead to very impractical contexts. Especially in the case of highly fragmented genomes, where if the --draft flag is not specified by human error, GenoVi would render multiple, even hundreds of ideogram files. Even more impractical if a directory is given with multiple draft genomes. That is why we decided to force the user to make this a conscious and explicit decision.

Q9: I would call the output folder name "genovi", rather than the name of someone else's program.

A: Thank you for mentioning this. We changed the output folder name and files to genovi in the new version of the software.

Q10: consider support for gzipped genbank files

A: Thank you for the great suggestion. GenoVi now runs with gzipped genbank format files, which reduces user workload for processing multiple files without having to extract them manually. Also reducing the amount of storage needed for large sets of genomes.

II. Reviewer 2 (Dr. Vikash K Singh):

The authors have developed Python command-line application for the visualization circular genome representations of complete and draft genomes of bacteria and archaea. However, there are scope to impoverish the manuscript before acceptance.

Therefore, I recommend major revision as per the following suggestions.

Dear Dr. Vikash K. Singh,

We kindly appreciate your time reviewing our manuscript and software.
As stated, the previous version of the manuscript and software did not properly highlight the usefulness of GenoVi. To address the raised concerns by all the reviewers, we added new features to GenoVi that modified the accompanying manuscript.

To improve the scope of GenoVi, additional features were incorporated into the new version. This included: enabling directories as an input (analyzed either as *-s draft* or *-s complete*), processing all the files independently and creating compilation tables that combine the genomic features identified of each genome (general stats and COG categories); automatic generation of COG categories frequency per replicon or contig, and heatmaps that illustrates the abundance of COG categories identified. Additionally, the user can specify which COG categories should be displayed in the ideogram, rather by specifying directly the categories (individually or as a functional group), or by indicating the N most abundant categories. With these new features, GenoVi demonstrates to be useful in various settings, which modified the examples in the manuscript. Currently, the analysis of *Paraburkholderia* multipartite genomes was reduced in extension, and other examples were incorporated to better illustrate the versatility of GenoVi. To highlight GenoVi features in comparison with other tools, a comparative table was incorporated into the manuscript.

Q1. Authors should include a comparison table highlighting advantages of their new tool over already exiting tools.

A: As recommended by the reviewer, we have incorporated a new table (Table 1), comparing GenoVi with other tools currently available for genome visualization. The comparative table highlights the differences and unique features that our software incorporates.

Q2. I suggest to containerize their software using docker or singularity for portability across machine.

A: We thank you for this recommendation. Currently we are working to deliver more options for implementation of GenoVi, including a docker image file. As soon as this is achieved, we will update the corresponding wiki and documentation.

III. Reviewer 3:

It looks like the tool is useful for automatically creating circular genomics visualization. However, biggest concern I have is that the resulting circular visualizations themselves **do not seem to enable complete analysis, and many parts of the analysis in the Results and discussion section actually seem to rely on externally-generated visualization, such as heatmaps and box plots.**

Dear Reviewer 3,

Thank you for the dedicated revision of our manuscript and software. We are kindly grateful for all your comments and raised concerns about GenoVi.

As stated by your revision, the previous version of the manuscript and software did not properly highlight the usefulness of GenoVi. The main example focused on *Paraburkholderia* mostly used output tables for its analysis, which could be obtained by other software without the main participation of the visualization.

To address the raised concerns by all the reviewers, we added new features to GenoVi that modified the accompanying manuscript.

To improve the scope of GenoVi, additional features were incorporated into the new version. This included: enabling directories as an input (analyzed either as *-s draft* or *-s complete*), processing all the files independently and creating compilation tables that combine the genomic features identified of each genome (general stats and COG categories); automatic generation of COG categories frequency per replicon or contig, and heatmaps that illustrates the abundance of COG categories identified. Additionally, the user can specify which COG categories should be displayed in the ideogram, rather by specifying directly the categories (individually or as a functional group), or by indicating the N most abundant categories. With these new features, GenoVi demonstrates to be useful in various settings, which modified the examples in the manuscript. Currently, the analysis of *Paraburkholderia* multipartite genomes was reduced in extension, and other examples were incorporated to better illustrate the versatility of GenoVi.

Q1: - I wonder if the heatmaps and box plots illustrated in the manuscript can be generated automatically along with the circos plots. I think this will make the analysis process more seamless to users.

A: As stated by the reviewer, the illustration of heatmaps and box plots was an important part of the previous version of the manuscript. The new version of GenoVi integrates the automatic generation of heatmaps displaying the COG categories distribution in each replicon or contig of an input genome (Fig 6). Additionally there are some new features in GenoVi that will help the user visualize specific features in their genomes without further processing of the output tables (Fig 3 and Fig 4). Novel usage examples are included, which highlight the functionality of the software. This was included in documentation and wiki of GenoVi, as well as the sections “” of the manuscript.

Q2: - One of the main limitation of the circular visualizations in term of their interpretation is that it is difficult to see the COG distribution patterns accurately due to the use of large number of similar colors. For example, in Fig. 2A, it is really hard to find what are the

most representative COG categories. I wonder if it makes sense to enable users to filter COG categories, show only top-N categories, or etc.

A: Thank you for this great recommendation. As stated, COGs are hard to observe and identify due to the oversaturated visualization. This consideration was implemented in the new version of GenoVi, and now the user can select which COG categories to display, if specific ones are required the user can select just one (*--cogs Q*, **Fig 4**), as well as to display only the most abundant categories (top-N categories; *--cogs 5,* **Fig 3**). This allowed us to implement new examples of usage which are now part of the manuscript.

Q3: - Many color palettes seem to be not color-blindness friendly. I suggest using color-blindness friendly palette as a default one (e.g., https://colorbrewer2.org/ or https://mikemol.github.io/technique/colorblind/2018/02/11/color-safe-palette.html).

A: We thank the reviewer for commenting about this issue. As stated, almost none of the previous color palettes were color-blindness friendly. The new version of GenoVi includes five novel color-blindness friendly palettes (besides the previous 20 pre-fabricated palettes), and the default color palette is "Strong", a palette based on the suggested color-safe palette. All the figures of the manuscript were updated with the new five palettes (*-cs strong, dawn, blossom, autumn, paradise*)

Q4: - Sufficiently distinct colors for individual categories should be used whenever possible (e.g., currently, positive CDS, negative CDS, and rRNA are too similar which is difficult to differentiate in the visualization).

A: As stated by the reviewer, some of the previous palettes did not differentiate enough for a good analysis. The current version of GenoVi separated rRNA and tRNA to another track and corrected the color of genomic features in the new colour palettes to better differentiate these categories.

Q5: - Using the "complete" sequence option for the first tutorial example gives an error on my machine (i.e. "FileNotFoundError: [Errno 2] No such file or directory: 'circos-contig_1.png' -> 'circos/circos-contig_1.png'").
Having more test datasets in the repository would be valuable.

A: Thank you for raising this concern. We have checked GenoVi with different input files and found bugs that are now corrected. We encourage you to try the complete example again to check if everything works correctly. To complement this, more examples are now provided and uploaded here: https://zenodo.org/record/7331473. This information is now included in the manuscript.

Minor
Q6:- Color legends generated are sometimes too small to read.

A: Thank you for the input. We added this concern into the new version of GenoVi, and now the default size for the legend is easier to read.

Q7:- In general, I think interactive visualizations can make the analysis more effective (e.g., using Gos Python package, https://osf.io/yn3ce/), such as using mouse hover to see exact COG categories in tooltips.

A: We thank you for this information. We are looking forward to incorporate this feature in a future version of GenoVi. Additionally we are planning to develop a web version of GenoVi, where the interactive visualization will make the analysis more seamless for users.
