# Supplementary material for: GenoVi, an open-source automated circular genome visualizer for bacteria and archaea
Source: PLoS Comput Biol. 2023 Apr 4;19(4):e1010998. doi: 10.1371/journal.pcbi.1010998 (PMC10104344; doi:10.1371/journal.pcbi.1010998)
Supplement: Attachment — Submitted filename: Response to Reviewers.pdf [file pcbi.1010998.s007.pdf]

Response to Reviewers: PCOMPBIOL-D-22-01291

GenoVi, an open-source automated circular genome visualizer for bacteria and archaea

Andrés Cumsille, Roberto E. Durán, Andrea Rodríguez-Delherbe, Vicente Saona-Urmeneta, Beatriz Cámara, Michael Seeger, Mauricio Araya, Nicolás Jara, Carlos Buil-Aranda

I. Reviewer 1 (Dr. Michael J. Roach):

The authors have addressed our main concerns. The updated GitHub and user guide looks great!

Dear Dr. Michael J. Roach,

We are very grateful of your comments and dedicated revision of GenoVi and our manuscript. Your comments helped our manuscript to better highlight the potential of GenoVi.

Q1: Error in the Author summary: "difficulting its usability"

A: Thank you for pointing this error. We have changed the sentence to “hampering their usage” in the Author Summary section.

Q2: Ideas for future developments:

Consider support for alternative annotations to COGs, e.g. KEGG, Phrogs/pvogs for viral annotations etc.
Test dataset for Archaea

A: Thank you for this idea. It will be useful for unexperienced users to combine multiple tools for the annotation of genomes analyzed by GenoVi. Currently we want to focus on the interactive platform, to later implement other annotation tools and specialized databases like BacMet, MiBig and EAWAG.

We added the *Sulfolobos acidocaldarius* DG1 example to the input_test data on the github page. We will add an *Archaea* multiple genome example in the near future.

II. Reviewer 2 (Dr. Vikash K Singh):

The authors have taken into account the previous suggestions and remarks. The present manuscript is suitable for publication.

Dear Dr. Vikash K. Singh,

We thank you for your time reviewing our manuscript and software. We really appreciate all your observations and comments.

III. Reviewer 3 (Dr. Sehi L'Yi):

Thank you for making changes. I do not have further comments.

Dear Reviewer 3,

Thank you for the revision of our manuscript and software. We are kindly grateful for all your feedback that lastly improved GenoVi.
