# Supplementary material for: GenoVi, an open-source automated circular genome visualizer for bacteria and archaea
Source: PLoS Comput Biol. 2023 Apr 4;19(4):e1010998. doi: 10.1371/journal.pcbi.1010998 (PMC10104344; doi:10.1371/journal.pcbi.1010998)
Supplement: Attachment — Submitted filename: Vikash_review_PCOMPBIOL-D-22-01291.docx [file pcbi.1010998.s005.docx]

The authors have developed Python command-line application for the visualization circular genome representations of complete and draft genomes of bacteria and archaea. However, there are scope to impoverish the manuscript before acceptance.

Therefore, I recommend major revision as per the following suggestions.

1. Authors should include a comparison table highlighting advantages of their new tool over already exiting tools.

2. I suggest to containerize their software using docker or singularity for portability across machine.
